# Influence of Antithrombotics on Severely Injured Patients Over 50 With Blunt Abdominal Trauma

**DOI:** 10.1007/s00068-026-03196-3

**Published:** 2026-05-27

**Authors:** Nicolas Eibinger, Belinda Limberger, Nina Hörlesberger, Paul Puchwein, Rolf Lefering, Tobias Peter Bayer, Jonin Serafin Zumsteg, Hans-Christoph Pape, Kai Oliver Jensen

**Affiliations:** 1 Department of Orthopaedics and Trauma, Medical University of Graz, Auenbruggerplatz 5, Graz, 8036 Austria; 2 Department of Trauma, University Hospital Zurich, Zurich, Switzerland; 3 Institute for Research in Operative Medicine (IFOM), University Witten/Herdecke, Cologne, Germany

**Keywords:** Abdominal trauma, Blunt trauma, Geriatic trauma, Antithrombotic medication, Mortality risk

## Abstract

**Introduction:**

Severe abdominal trauma represents a critical subset of injuries, particularly in the aging European population, where the prevalence of preexisting anticoagulant or antiplatelet therapy is increasing. While the impact of such medications on outcomes in traumatic brain injury is well studied, limited data exist regarding their influence in patients with abdominal trauma.

**Materials and methods:**

This retrospective cohort study used data from the TraumaRegister DGU^®^ between January 2015 and December 2023. Inclusion criteria were patients aged ≥ 50 years with severe blunt abdominal trauma (AIS Abdomen ≥ 3) without relevant head injury (AIS Head ≤ 3) from Austria, Germany, or Switzerland. Patients were grouped according to pre-injury antithrombotic medication (no medication [NM] vs. antithrombotic medication [AM], with subgroups). Statistical analysis included descriptive comparisons, standardized mortality ratios (SMR) using RISC III, and multivariate logistic regression to identify independent predictors of hospital mortality.

**Results:**

Of 328,281 patients, 4,069 met inclusion criteria (2,831 NM; 1,238 ATM). Patients in the AM group were older (74.1 vs. 62.3 years) and had higher mortality (25.0% vs. 10.4%), particularly in the DOAC subgroup (30.3%). SMR analysis demonstrated increased mortality in the AM group compared to expected rates. In multivariate logistic regression, antithrombotic medication remained an independent predictor of mortality (OR 1.28, 95% CI 1.03–1.58; *p* = 0.025). Prehospital management differed significantly, with lower intubation and tranexamic acid use in the AM group. Surgical management was largely comparable.

**Conclusion:**

Antithrombotic therapy is independently associated with increased mortality in patients aged ≥ 50 years with severe abdominal trauma. In addition to distinct treatment patterns and possible undertriage, these findings highlight the need for tailored trauma management strategies in this high-risk population.

## Introduction

Severe trauma is the leading cause of disability and death worldwide. Abdominal injuries occur in less than 10% of all trauma patients, but severe abdominal injuries are found in up to 30% of these patients [1–3]. The majority of abdominal traumas in Europe are caused by blunt mechanisms [4].

As the global population ages, the proportion of individuals aged ≥ 65 years in Europe is expected to reach at least 30% by 2050, which means that the number of elderly people with severe abdominal trauma will also increase [5]. Currently, the number of trauma patients older than 60 years has increased by approximately 1.6% per year in industrial nations [6]. The same trend was observed for the number of people receiving ongoing anticoagulant or antiplatelet medications [7, 8].

While the effect of antiplatelet or anticoagulant drugs on mortality in patients with traumatic brain injury is often described in the literature, less data are available for patients with abdominal trauma [5, 9, 10].

The main goal of this study was to analyse the effect of pre-trauma antiplatelet or anticoagulant drug intake in patients with severe abdominal trauma.

## Methods

The TraumaRegister DGU^®^ of the German Trauma Society (Deutsche Gesellschaft für Unfallchirurgie, DGU) was founded in 1993. The aim of this multi-centre database is a pseudonymized and standardized documentation of severely injured patients.

Data are collected prospectively in four consecutive time phases from the site of the accident until discharge from hospital: (A) Prehospital phase, (B) Emergency room and initial surgery, (C) Intensive care unit (ICU) and (D) Discharge. The documentation includes detailed information on demographics, injury pattern, comorbidities, pre- and in-hospital management, course on intensive care unit, relevant laboratory findings including data on transfusion and outcome of each individual. The Inclusion criterion is admission to hospital via emergency room with subsequent ICU/ICM care or reach the hospital with vital signs and die before admission to ICU.

The infrastructure for documentation, data management, and data analysis is provided by AUC – Academy for Trauma Surgery (AUC - Akademie der Unfallchirurgie GmbH), a company affiliated to the German Trauma Society. The scientific leadership is provided by the Committee on Emergency Medicine, Intensive Care, and Trauma Management (Sektion NIS) of the German Trauma Society. The participating hospitals submit their data pseudonymized into a central database via a web-based application. Scientific data analysis is approved according to a peer review procedure laid down in the publication guideline of TraumaRegister DGU^®^.

The participating hospitals are primarily located in Germany (90%), but a rising number of hospitals of other countries contribute data as well (at the moment from Austria, Belgium, China, Finland, Luxembourg, Slovenia, Switzerland, The Netherlands, and the United Arab Emirates). Currently, about 38,000 cases from almost 700 hospitals are entered into the database per year.

Participation in the TraumaRegister DGU^®^ is voluntary. For hospitals associated with TraumaNetzwerk DGU^®^, however, the entry of at least a basic dataset is obligatory for reasons of quality assurance.

The present study is in line with the publication guidelines of the TraumaRegister DGU^®^ (TR-DGU) and is registered as TR-DGU project ID TR-DGU 2024-026.

In the TR-DGU, all injuries are coded based on the Abbreviated Injury Scale (Abbreviated Injury Scale (AIS), version 2005; 2008 update) [11].

The Injury Severity Score (ISS) was used [12].

Coagulopathy is defined in TraumaRegister DGU^®^ by the following values: PTT ≥ 40 s, INR ≥ 1.4, or Quick ≤ 60, which corresponds to the Berlin polytrauma definition [13].

### Study population

All data was collected from the TraumaRegister DGU^®^.

#### Inclusion criteria

All patients aged ≥ 50 years who had suffered from a blunt abdominal injury with an AIS severity ≥ 3 were included. Patients with relevant head injuries (AIS ≥ 3) were excluded. Patients were required to have been treated in Austria, Germany, or Switzerland between 01/2015 and 12/2023.

#### Exclusion criteria

All patients with missing documentation of coagulation medication and those who were transferred to another hospital within the first 48 h were excluded. Patients with penetrating trauma were excluded from the study.

For further analysis, patients were divided into the following two groups with four subgroups for group two:

1) No antithrombotic medication (NM).

2) Antithrombotic medication (AM)

2a) Antiplatelet drug (APD).

2b) Vitamin K antagonist (VKA).

2c) Direct oral anticoagulants (DOACs).

2d) Heparinoids.

### Statistical analysis

Statistical analyses were performed using SPSS statistical software (SPSS Version 29 (IBM Inc., Armonk, NY, USA). Data are presented as mean ± standard deviation (SD) and as percentages for categorical variables. Patients receiving ongoing heparin therapy were excluded from the subgroup analysis because of the small sample size. Standardized mortality ratios (SMR) were calculated as observed divided by expected mortality rates, where the Revised Injury Severity Classification (RISC) score was used to estimate the expected mortality (version III). Version III of the RISC score uses the same predictors as RISC II [14] but with updated coefficients; it was developed and validated on the most recent data from TR-DGU [15]. Outcome prediction using RISC was valid only for the primary admitted cases. SMRs are presented with a 95% confidence interval (CI95) which was calculated based on the respective CI95 of the mortality rates. In order to further evaluate the effect of AM, a logistic regression analysis was performed with hospital mortality as dependent variables. The following predictors were considered besides the AM: age, sex, Injury Severity Score (ISS), pre-injury health status (ASA), suspected suicide, inter-hospital transfer, blood transfusion, and polytrauma (according to the Berlin definition: at least two body regions together with a physiological problem: unconsciousness, or hypotension, or acidosis, or coagulopathy, or high age). Results are presented as odds ratios (OR) with 95% confidence intervals (CI).

Due to multiple comparisons, formal statistical testing was performed in selected comparisons using only the chi-square test. The level of significance was set at *p* < 0.05.

## Results

**Fig. 1:**
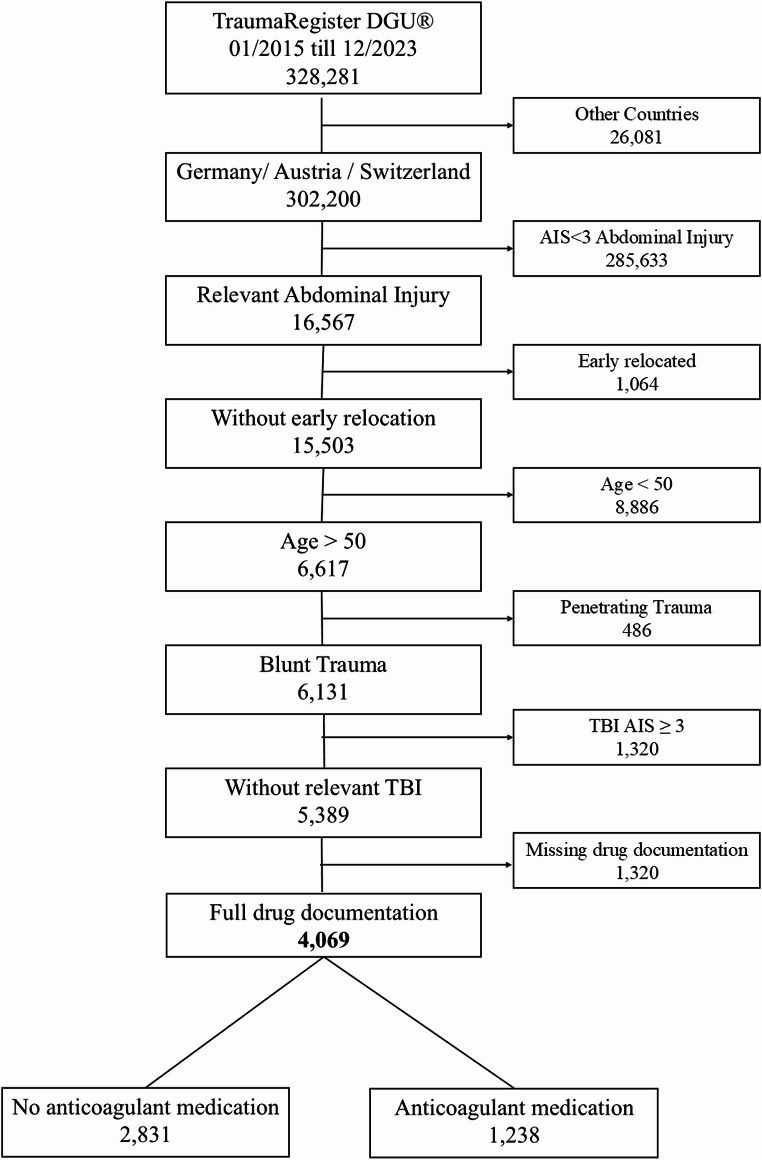
Study outline

A total of 328,281 patients were documented at the TraumaRegister DGU^®^ between January 2015 and December 2023. Of these patients, 4,069 from 577 hospitals met all the inclusion criteria. In total 2,831 were included in the NM group and 1,238 in the AM group, respectively. Only 19 patients who were undergoing ongoing heparin therapy were identified (Fig. 1).

The mean age (SD) in the NM group was 62.3 (9.7) years and 74.1 (10.4) (*p* < 0.001) in the medication group (AM). The cohort with intake of DOACs contained older patients with an average of 76.6 (9.5) years. The mean ISS in the NM group was 27.7 (12.3) and 26.5 (11.5), group (*p* = 0.016). In the AM group Relevant extremity injuries were significantly less frequent than in the NM group (31.5% vs. 24.2%, *p* < 0.001). No significant difference in thoracic injuries was observed between the two groups (*p* = 0.077). Liver injuries were significantly more common in the NM group (20.8% vs. 13.8%, *p* < 0.001). Injuries to the spleen were documented in 43.8% and 43.7% of patients in the NM and AM groups, respectively (Table 1).

The most common cause of injury in the NM group was car accidents (31.1%), followed by motorcycle accidents (18.4%) and falls above three meters (13.4%). Low falls below three meters (31.3%), car accidents (29.0%), and high falls above three meters (10.6%) were the main causes of injuries in the AM group (Table 2).

The Prehospital intubation rates were 17.2% (NM) vs. 11.8% (AM) (*p* < 0.001). Tranexamic acid was applied in 19.0% in the NM group and 13.2% in the NM and AM group, respectively (*p* < 0.001). The percentage of patients with a prehospital systolic blood pressure ≤ 90 mmHg were 17.1 (NM) and 23.4 (AM) (*p* < 0.001) (Table 1).

**Table 1:** Baseline data

	NM	AM	APD	VKA	DOAC
Cases	2,831	1,238	663	193	363
Age (Mean, SD)	62.3 (9.7)	74.1 (10.4)*	72.4 (10.7)	75.3 (9.8)	76.6 (9.5)
Male (%)	1,853 (65.5)	837 (67.6)	454 (68.5)	131 (67.9)	242 (66.7)
ISS (Mean, SD)	27.7 (12.3)*	26.5 (11.5)	26.6 (11.3)	26.5 (12.2)	26.4 (11.4)
Thorax AIS ≥ 3 (%)	1730 (61.1)	720 (58.2)	386 (58.2)	117 (60.6)	203 (55.9)
Extremity AIS ≥ 3 (%)	892 (31.5)	300 (24.2)*	171 (25.8)	46 (23.8)	78 (21.5)
Liver injury (%)	588 (20.8)	171 (13.8)*	92 (13.9)	24 (12.4)	54 (14.9)
Spleen injury (%)	1,241 (43.8)	541 (43.7)	295 (44.5)	84 (43.5)	153 (42.1)
Kidney injury (%)	443 (15.6)	180 (14.5)	83 (12.5)	31 (16.1)	63 (17.4)
Injury mechanism					
Car (%)	866 (31.1)	351 (29.0)	201 (30.8)	55 (28.6)	91 (26.1)
Motorcycle (%)	514 (18.4)	113 (9.3)*	68 (10.4)	18 (9.4)	25 (7.2)
Bicycle (%)	279 (10.0)	91 (7.5)	56 (8.6)	8 (4.2)	26 (7.4)
Pedestrian (%)	142 (5.1)	65 (5.4)	42 (6.4)	6 (3.1)	16 (4.6)
High Fall >3 m (%)	373 (13.4)	127 (10.6)	69 (10.6)	21 (10.9)	37 (10.6)
Low Fall <3 m (%)	310 (11.1)	379 (31.3)*	163 (25.0)	75 (39.1)	132 (37.8)
Other	302 (10.8)	84 (6.9)	53 (8.1)	9 (4.7)	22 (6.3)
Prehospital data					
Intubation (%)	413 (17.2)	119 (11.8)*	75 (13.5)	20 (12.6)	23 (8.2)
iv Fluids (Mean, SD)	752 (642)	685 (563)	687 (566)	687 (588)	677 (524)
Tranexamic Acid (%)	443 (19.0)	128 (13.2)*	71 (13.1)	24 (15.9)	33 (12.2)
SBP ≤ 90 mmHg (%)	373 (17.1)	211 (23.4)*	98 (20.0)	34 (23.3)	77 (30.1)
Hospital					
SBP ≤ 90mmHg ER (%)	473 (17.8)	246 (21.5)*	128 (20.7)	40 (22.1)	74 (22.4)
Coagulopathy (%)	332 (12.2)	452 (38.0)*	111 (17.5)	164 (87.7)	170 (48.6)
Surgery rate (%)	2,375 (83.9)	994 (80.3)*	545 (82.2)	147 (76.2)	289 (79.6)
Emergency surgery (%)	1,495 (52.8)	621 (50.2)	336 (50.7)	104 (53.9)	176 (48.5)
pRBC (%)	783 (27.8)	424 (34.3)*	215 (32.5)	72 (37.3)	133 (36.7)
pRBC > 10 (%)	105 (3.7)	59 (4.8)	35 (5.3)	10 (5.2)	14 (3.9)
PPSB (%)	185 (9.5)	175 (21.3)*	56 (12.6)	57 (50.9)	60 (23.4)
Mortality rate (%)	294 (10.4)	309 (25.0)*	142 (21.4)	51 (26.4)	110 (30.3)
Died within 24 h (%)	146 (5.2)	129 (10.4)*	59 (8.9)	18 (9.3)	48 (13.2)
Length of ICU stay (d)	5 (2–12)	5 (2–13)	5 (2–13)	6 (2–15)	6 (2–12
Length of Hospital stay (d)	16 (9–28)	15 (8–26)*	15 (8–27)	15 (7–27)	14 (8–24)

Length of ICU and hospital stay are shown as median and quartiles. * *p* < 0.05 for NM versus AM; NM = no antithrombotic medication, AM = antithrombotic medication, ISS = Injury severity score; SBP = systolic blood pressure, pRBC = packed red blood cells, d = days, ER = emergency room

The overall surgery rates were significantly lower in the AM group than in the NM group (80.3% vs. 83.9%, *p* = 0.005), whereas no significant difference in emergency surgeries was found (50.2% vs. 52.8%, *p* = 0.12). Patients in the AM group were significantly more likely to receive pRBCs (34.3% vs. 27.8%, *p* < 0.001) and PPSBs (21.3% vs. 9.5%, *p* < 0.001). No significant difference was observed in the mass transfusion of pRBCs (*p* = 0.12).

The average length of hospital stay was significantly shorter in the AM group than in the NM group (15 vs. 16 days, *p* = 0.001).

The observed mortality rates were 10.4% (NM) and 25.0% (AM), respectively. The highest mortality rate (30.3%) was observed in the DOAC group. The SMR based on RISC III for the AM group showed a significantly higher mortality rate than expected (SMR 1.12, 95% CI 1.01–1.24), whereas this was not observed in the NM group (SMR 1.03, 95% CI 0.91–1.14). The details are listed in Table 2.

**Table 2:** Expected and observed mortality rates in primary admitted cases

	NM	AM	APD	VKA	DOAC
All cases	2,831	1,238	663	193	363
Primary admitted	2,511	1,059	580	165	301
Observed mortality	265 (10.6%)	273 (25.8%)	126 (21.7%)	46 (27.9%)	95 (31.6%)
95% CI for observed mortality	9.4–11.8	23.1–28.4	18.4–25.1	21.0–34.7	26.3–36.8
Expected mortality based on RISC III	10.3%	23.0%	19.1%	28.6%	27.0%
SMR with 95% CI	1.03 0.91–1.14	1.12 1.01–1.24	1.14 0.91–1.14	0.97 0.74–1.21	1.17 0.97–1.36

No antithrombotic medication (NM), antithrombotic medication (AM), Antiplatelet drugs (APD), Vitamin K antagonists (VKA), Direct oral anticoagulants (DOACs)

The only significant difference in the surgical management of abdominal trauma between the NM and AM groups was found in injuries graded with AIS 3. Conservative treatment was significantly more common at the AM group (27% vs. 20%, *p* < 0.001). Emergency laparotomy was performed significantly more frequently in the NM group (29% vs. 23%, *p* = 0.017), whereas emergency embolization was significantly more common in the AM group (5% vs. 7%, *p* = 0.037). The details are listed in Table 3.

**Table 3:** Surgical management in patients with and without antithrombotic medication, depending on the severity of abdominal trauma; 3 cases with AIS 6 excluded. # emergency intervention before ICU admission

	AIS 3		AIS 4		AIS 5	
	NM *n* = 1629	AM *n* = 653	NM *n* = 947	AM *n* = 456	NM *n* = 253	AM *n* = 128
Conservative	334 (20%)	177 (27%)*	89 (10%)	57 (13%)	23 (9%)	10 (8%)
Any surgery	1295 (80%)*	476 (73%)	849 (90%)	399 (87%)	230 (91%)	118 (92%)
Abdominal surgery	1008 (62%)	381 (58%)	786 (83%)	378 (83%)	226 (89%)	116 (91%))
- Laparotomy #	440 (29%)*	145 (23%)	463 (51%)	215 (50%)	155 (65%)	86 (72%)
- Embolization #	75 (5%)	44 (7%)*	67 (7%)	42 (10%)	6 (3%)	5 (4%)

No antithrombotic medication (NM), antithrombotic Medication (AM)

Multivariate logistic regression analysis including AM as a yes/no predictor showed a significantly increased risk of death after adjustment: OR 1.28 (95% CI 1.03–1.58, *p* = 0.025) (Table 4).

**Table 4:** Results of logistic regression analysis with hospital mortality as dependent variable and antithrombotic medication as yes/no

Predictor	Value	OR	05%-CI for OR	*p*-value
ISS	Per point	1.061	1.054–1.069	< 0.001
Age (ref: 50–59)	60–69 70–79 80–89 > 90	1.25 2.00 5.75 11.31	0.96–1.61 1.53–2.63 4.28–7.71 6.74–18.99	0.094 < 0.001 < 0.001 < 0.001
Pre-injury ASA (ref: 1/2)	3/4	1.96	1.60–2.39	< 0.001
Sex (ref: females)	males	1.31	1.09–1.58	0.005
Suspected suicide (ref: no)	yes	2.52	1.83–3.47	< 0.001
Inter-hospital transfer (ref: primary admission)	yes	0.67	0.51–0.88	0.004
Polytrauma (ref: no)	yes	1.51	1.21–1.89	< 0.001
Blood transfusion (ref: no)	yes	1.76	1.47–2.11	< 0.001
Antithrombotic drugs (ref: no)	yes	1.28	1.03–1.58	0.025

Ref = reference category; OR = odds ratio; CI = confidence interval

## Discussion

In an aging society, the rising number of geriatric trauma patients is a major challenge in healthcare. These patients require urgent specialized care [16]. Due to better medical treatment, increased life expectancy, and a higher level of activity and mobility, the number of severely injured patients above 50 years of age has increased over the last few years [17].

The purpose of this study was to assess the role of antiplatelet and anticoagulant drugs in the treatment and outcome of severely injured patients aged ≥ 50 years with severe abdominal trauma.

Our main results are:

1. Different injury mechanisms and prehospital treatments in the NM and AM groups due to the higher age in the AM group.
2. Significantly higher SMR in the AM group.
3. After adjusting for several confounders antithrombotic therapy was independently associated with increased mortality.
4. No relevant differences in the treatment of the abdominal injuries.

In our data, we observed an obvious difference in the injury mechanisms between the NM and AM groups. While car accidents are comparable (31.1% vs. 29.0%) the percentage of motorcycle accidents differs from 18.4% (NM) to 9.3% (AM). Low falls (< 3 m) occurred more frequently in the AM group (31.3%) than in the NM group (10.9%). The main reason for this might be the increased age of the patients in the AM group (62.3 y vs. 74.1 y). Changes in injury mechanisms with increasing age have also been observed in several other studies [17–20].

We found several differences in prehospital management of the AM group compared to the NM group. Although the overall ISS was comparable, there was a significantly lower intubation rate in the AM group (11.8% vs. 17.2% in the NM group). In addition, the percentage of patients treated with tranexamic acid was significantly lower in the AM group (13.2% vs. 19.0% in NM), even though a BP < 90 mmHg was detected in significantly more cases (23.4% vs. 17.1% in NM). These findings may reflect a combination of factors, including undertriage of elderly patients, physician-dependent decision-making, and limitations of existing triage protocols in older populations. Furthermore, the registry does not capture the clinical reasoning behind prehospital decisions, which limits interpretation. Undertriage of elderly patients is a well-known problem in the literature and might also influence the overall survival of these patients [17, 21–23]. A reason for the rare use of tranexamic acid might be the consideration of potential contraindications, such as a history of stroke, peripheral vascular disease, thromboembolic disease, or vascular stent, which are also the main indications for antithrombotic therapies [24]. 

The observed mortality rate in the AM group was more than twice that in the NM group (25.0% vs. 10.4%). Several other studies have shown that increasing age of severely injured patients is associated with higher mortality rates [5, 6, 17, 19, 25, 26]. In our patient cohort, the SMR calculated with the new RISC III, was significantly increased in the AM group. The RISC III model incorporates updated coefficients based on recent registry data, including age-related effects, suggesting that the observed difference is not solely explained by age.

One reason for this might be the comorbidities of patients undergoing ongoing blood dilutive therapies or the therapy itself. Beyond this, the multivariate logistic regression analysis demonstrated that antithrombotic therapy remained independently associated with mortality after adjustment for relevant confounders, including age, injury severity, comorbidities, transfusion requirements, and polytrauma. This suggests that the observed effect is not solely explained by baseline differences between groups but may reflect an independent impact of antithrombotic or factors closely linked to its use. Similar associations have been described in patients with traumatic brain injury, where antithrombotic therapy has been shown to adversely affect outcomes [9, 10]. Another factor might be the withdrawal of life-sustaining therapy in older patients (WLST). This topic is heavily discussed in the literature and may lead to higher overall mortality rates [27, 28].

Regarding surgical management, overall intervention rates were similar between groups. However, we observed a higher rate of conservative treatment and embolization in patients receiving antithrombotic medication, suggesting a shift toward less invasive management strategies. This may reflect clinical caution due to bleeding risk or differences in patient characteristics. Significant differences were observed only in injury grades of AIS 3. These results were similar to those reported by Bhattacharya et al. [29]. They did not find any difference in the surgery rates for liver and splenic injuries in patients with and without antithrombotic medication. Overall, the surgery rates for abdominal trauma in our patient group were relatively high compared to those reported in the literature. For example, Renzulli et al. reported a surgery rate of only 22.8% for abdominal injuries [30]. In general, laparotomy rates have decreased over the last few years, and nonoperative management and angioembolization are becoming more popular [4, 31]. Ferrah et al. showed that laparotomy rates decreased from 60% in 2007 to 44% in 2016, and angioembolisation rates increased from 6 to 20% at the same time [32].

It is important to recognize the limitations of this study. Owing to the retrospective nature of the study, the findings indicate associations rather than causality. Registry data lack detailed information on medication specifics, such as timing of last intake, dosage, or reversal strategies. In addition, decision-making processes in prehospital and in-hospital settings are not captured. Residual confounding and selection bias cannot be excluded. The TraumaRegister DGU^®^ is renowned for its high-quality data. For further sub-analyses, the number of patients was too small because severe abdominal trauma is uncommon.

## Conclusion

Severely injured elderly patients will represent a major challenge in future trauma care. In addition to existing comorbidities, the use of antiplatelet and anticoagulant drugs further complicates the management of this patient population.

Our data demonstrate an association between antithrombotic therapy and increased mortality in patients aged ≥ 50 years with severe abdominal trauma, which remains significant even after adjustment for relevant confounders. Furthermore, we observed differences in prehospital management, suggesting possible undertriage, likely related to advanced age and low-energy trauma mechanisms.

No clinically relevant differences were identified in the surgical management of abdominal injuries. Further investigations are needed to better understand the underlying mechanisms and to optimize prehospital and in-hospital treatment strategies for this vulnerable patient group.

## Data Availability

No datasets were generated or analysed during the current study.
